# NAC Candidate Gene Marker for *bgm-1* and Interaction With QTL for Resistance to *Bean Golden Yellow Mosaic Virus* in Common Bean

**DOI:** 10.3389/fpls.2021.628443

**Published:** 2021-03-25

**Authors:** Alvaro Soler-Garzón, Atena Oladzad, James Beaver, Stephen Beebe, Rian Lee, Juan David Lobaton, Eliana Macea, Phillip McClean, Bodo Raatz, Juan Carlos Rosas, Qijian Song, Phillip N. Miklas

**Affiliations:** ^1^Irrigated Agriculture Research and Extension Center, Washington State University, Prosser, WA, United States; ^2^Department of Plant Sciences, North Dakota State University, Fargo, ND, United States; ^3^Department of Agroenvironmental Sciences, University of Puerto Rico, Mayagüez, Puerto Rico; ^4^Bean Program, Agrobiodiversity Area, International Center for Tropical Agriculture (CIAT), Cali, Colombia; ^5^School of Environmental and Rural Sciences, University of New England, Armidale, SA, Australia; ^6^Department of Agricultural Engineering, Zamorano University, Zamorano, Honduras; ^7^Soybean Genomics and Improvement Laboratory, United States Department of Agriculture – Agricultural Research Service (USDA-ARS), Beltsville, MD, United States; ^8^Grain Legume Genetics and Physiology Research Unit, United States Department of Agriculture – Agricultural Research Service (USDA-ARS), Prosser, WA, United States

**Keywords:** frameshift mutation, geminivirus, genome-wide association study, marker-assisted selection, *Phaseolus vulgaris*

## Abstract

Genetic resistance is the primary means for control of *Bean golden yellow mosaic virus* (BGYMV) in common bean (*Phaseolus vulgaris L.*). Breeding for resistance is difficult because of sporadic and uneven infection across field nurseries. We sought to facilitate breeding for BGYMV resistance by improving marker-assisted selection (MAS) for the recessive *bgm-1* gene and identifying and developing MAS for quantitative trait loci (QTL) conditioning resistance. Genetic linkage mapping in two recombinant inbred line populations and genome-wide association study (GWAS) in a large breeding population and two diversity panels revealed a candidate gene for *bgm-1* and three QTL BGY4.1, BGY7.1, and BGY8.1 on independent chromosomes. A mutation (5 bp deletion) in a NAC (No Apical Meristem) domain transcriptional regulator superfamily protein gene *Phvul.003G027100* on chromosome Pv03 corresponded with the recessive *bgm-1* resistance allele. The five bp deletion in exon 2 starting at 20 bp (Pv03: 2,601,582) is expected to cause a stop codon at codon 23 (Pv03: 2,601,625), disrupting further translation of the gene. A T_*m*_-shift assay marker named PvNAC1 was developed to track *bgm-1*. PvNAC1 corresponded with *bgm-1* across ∼1,000 lines which trace *bgm-1* back to a single landrace “Garrapato” from Mexico. BGY8.1 has no effect on its own but exhibited a major effect when combined with *bgm-1*. BGY4.1 and BGY7.1 acted additively, and they enhanced the level of resistance when combined with *bgm-1*. T_*m*_-shift assay markers were generated for MAS of the QTL, but their effectiveness requires further validation.

## Introduction

*Bean golden yellow mosaic virus* (BGYMV), first reported more than 50 years ago (Gálvez and Morales, 1989; Morales and Anderson, 2001), continues to plague common bean (*Phaseolus vulgaris* L.) production in Central America and the Caribbean (Chang-Sidorchuk et al., 2018). This bipartite DNA geminivirus species belongs to the genus *Begomovirus* (family Geminiviridae) and is closely related to the *Bean golden mosaic virus* (BGMV) species. BGYMV is sap transmitted and prominent in Central America and Caribbean countries. BGMV, the first described member of this genus, is found in South America and is not sap transmitted through mechanical inoculation. For both species, the *Bemisia tabaci* (Gennadius) whitefly acts as a vector. BGYMV epidemics are influenced by prevalence of the whitefly vector in the field in the early stages of crop development. Severe BGYMV epidemics can result in up to 100% crop loss (Gálvez and Morales, 1989; Morales and Anderson, 2001).

For common beans, combining host resistance with appropriate cropping system practices that reduce the prevalence of the vector is a critical strategy for controlling BGYMV (Coyne et al., 2003). Genetics and breeding research on BGYMV resistance (Beebe and Corrales, 1991; Miklas et al., 2006; Blair and Morales, 2008; Singh and Schwartz, 2010; Beaver et al., 2020a) shows that intra- and inter-racial crosses have combined resistances from diverse sources to generate materials with more effective resistance (Morales and Niessen, 1988; Beebe and Corrales, 1991; Morales and Singh, 1993; Singh et al., 2000). To further expand the diversity of resistance, interspecific crosses were used to transfer resistance from *P. coccineus* (Osorno et al., 2007).

Genetic studies discovered independent genes *bgm-1* (Blair and Beaver, 1993), *bgm-2* (Velez et al., 1998), and *bgm-3* (Osorno et al., 2007) that condition high levels of resistance against leaf yellowing, mosaic, and chlorosis. *Bgp* (Acevedo-Román et al., 2004) and *Bgp-2* (Osorno et al., 2007) were found to confer resistance to pod deformation, while a QTL on Pv04 [renamed here BGY4.1^*DX*^ using QTL nomenclature guidelines (Miklas and Porch, 2010)] and Pv07 (BGY7.1^*DX*^) contributed to delayed/reduced leaf chlorosis (Miklas et al., 1996).

*bgm-1* is the most widely used gene in breeding for resistance, in part because of its Middle American origin, but also because molecular markers R2 RAPD (random amplified polymorphic DNA) (Urrea et al., 1996) and the subsequent SR2 SCAR (sequence characterized amplified sequence) (Blair et al., 2007) have been used since 1995 to mobilize the gene into Central American and Caribbean germplasm. A SCAR marker SW12 (Singh et al., 2000), linked to QTL BGY4.1, has increased the deployment of the QTL in combination with *bgm-1*. Although lines with *bgm-1* and BGY4.1 have little to no leaf chlorosis, they suffer significant pod deformation and associated yield loss unless they are combined with other resistance QTL or genes such as *Bgp*. However, markers linked to the additional QTL or *Bgp* have not been developed. Therefore, it is necessary to use field nurseries with moderate to severe BGYMV infection to detect lines with high levels of resistance. *bgm-2* is deployed in only a few lines and underutilized because a linked marker is not available for the gene. Similarly, *bgm-3* and *Bgp-2* are unmapped, and their recent transfer from *P. coccineus* into dry bean has limited their use. However, this BGYMV resistance of diverse origin may allow bean breeders to better respond to the emergence of strains virulent to *bgm-1.* Host candidate genes for the multiple BGYMV genetic resistance factors have not been discovered, although many geminivirus-host plant protein interactions are known (Hanley-Bowdoin et al., 2013). Mutant host factors involved in these interactions would be reasonable candidate genes to pursue. For example, a NAC (no apical meristem) domain transcriptional regulator superfamily protein (SlNAC1) was found to affect geminivirus replication in tomato (Selth et al., 2005). Subsequently, a recessive gene *ty-1* conditioning resistance to *Tomato leaf curl virus* (a geminivirus) was associated with SlNAC1 (Hutton et al., 2012).

To generate germplasm with the most effective resistance, the current breeding strategy is to use MAS for *bgm-1* and BGY4.1 followed by phenotypic selection for the most resistant materials to combine *Bgp* and additional QTL. This strategy has been difficult to implement recently because disease pressure was sporadic throughout Central America and the Caribbean region during the last decade, making it difficult to identify high levels of resistance. But now, as disease occurrence and severity of BGYMV epidemics is increasing once again in the region, perhaps as a result of climatic events, many recently developed cultivars and breeding materials with supposed BGYMV resistance have been exposed as susceptible, moderately susceptible, or segregating for resistance. Dependence on *bgm-1* without selection for other factors complementing *bgm-1* has contributed to the problem. Moreover, the SR2 SCAR marker, by today’s standards, is not tightly linked to *bgm-1* (∼3–7 cM), which results in selection of a few lines that possess the marker but nonetheless are susceptible to the pathogen. Furthermore, confidence in MAS for BGY4.1 using SCAR SW12 is low because the QTL was originally mapped with a broad confidence interval defined by few markers (Miklas et al., 1996).

The recent release of common bean genomic tools, including multiple *P. vulgaris* reference genome assemblies of Chaucha Chuga (G19833) (Schmutz et al., 2014), BAT 93 (Vlasova et al., 2016), OAC-REX (Perry et al., 2013), and UI-111^1^; many resequenced genotypes (Lobaton et al., 2018); diversity panels with high numbers of SNPs generated by GBS or WGS (Cichy et al., 2015; Moghaddam et al., 2016; Wallace et al., 2018; Oladzad et al., 2019); and BeadChip assays with 6,000–12,000 SNPs (Song et al., 2015; Miklas et al., 2020), significantly increased the capacity to obtain tightly linked markers for target genes. Obtaining improved markers linked to rust (Hurtado-Gonzales et al., 2017) and *Bean common mosaic virus* (BCMV) (Bello et al., 2014) resistance genes was facilitated by these new genomic tools. Vasconcellos et al. (2017) improved resolution of previously mapped QTL conditioning resistance to white mold (*Sclerotinia sclerotiorum*) by re-genotyping RIL populations with the BARCBean6K_3 BeadChip with 5398 SNP markers (Song et al., 2015) to increase marker density. Other white mold QTL have been fine-mapped using bulk introgression that depends upon the reference genomes for marker discovery (Mamidi et al., 2016).

To increase the effectiveness of breeding for BGYMV resistance, we have sought to improve MAS for *bgm-1* and the BGY4.1 QTL, and to expand efforts to identify new QTL for resistance. Specific objectives were to: (i) fine map *bgm-1* for candidate gene discovery; (ii) narrow the genomic intervals for the BGY4.1 and BGY7.1 QTL; and (iii) search for new QTL conditioning resistance to BGYMV using linkage mapping and GWAS in segregating recombinant inbred and advanced breeding line populations, and in diversity panels comprised of Central American bean germplasm.

## Materials and Methods

### Toward Candidate Gene Markers for *bgm-1*

Candidate genes for the *bgm-1* region conditioning BGYMV resistance were targeted by resequencing select lines and GWAS mapping in different segregating populations. GBS data for lines DOR 476, SCR 9, SCR 16, and Tio Canela 75 with the resistance *bgm-1* allele and lines CAL 96, SCR 2, SEN 56, SEA 5, and SMC 33 with the *Bgm-1* susceptible allele were compared using the NGSEP pipeline (Perea et al., 2016; Lobaton et al., 2018) with G19833 v2.1^2^ as the *P. vulgaris* reference genome. These lines are parents of a MAGIC (Multi-parent Advanced Generation Inter-Crosses) population generated at CIAT (Diaz et al., 2019). Allele-specific primers were designed according to Wang et al. (2005) using the software Primer 3 (Untergasser et al., 2012) for SNP markers polymorphic between the *bgm-1* and *Bgm-1* genotypes. Fragments were amplified by PCR on an Eppendorf Mastercycler (Eppendorf AG, Hamburg, Germany) using a volume of 20 μl for PCR reaction containing 20 ng of genomic DNA, 1X Taq buffer, 1.5 mM MgCl_2_, 0.2 mM of dNTPs mix (Promega^®^, Madison, WI), 0.15 μM of each primer (two allele-specific forward primers and the common reverse primer), 1X EvaGreen (Biotium^®^), and 0.1 μL *Taq*1 polymerase (Promega^®^) under the following thermal profile: an initial denaturation step at 94°C for 2 min, then 38 cycles of denaturation at 92°C for 20 s, annealing for 20 s (the temperature was specific to each primer trio), and extension at 72°C for 20 s, and final extension at 72°C for 5 min. Melting point analysis for allele determination of the template DNA was performed with a fluorescence-detecting thermocycler (LightCycler^®^ 4890 Instrument II, Roche, Basal Switzerland) with EvaGreen fluorescent dye (Biotium^®^, Fremont, CA). Fluorescent detection by 1 min at 95°C and the melting curve step ramping from 65 to 95°C in increments of 1°C every 20 s.

The genomic region surrounding *bgm-1* was further narrowed in the DS RIL population (described below) with haplotyping using the polymorphic SNP markers identified above. A candidate gene within the narrowed *bgm-1* region was chosen for sequencing in three lines DOR 476, SCR 16, and Tio Canela 75 with the *bgm-1* and three lines SMC 33, SEL 1309, and DOR 364 with the *Bgm-1* allele. The candidate gene sequence from these six lines was aligned to four reference genome accessions G19833, BAT 93, OAC REX, and UI 111 (Perry et al., 2013; Schmutz et al., 2014; Vlasova et al., 2016)^3^. Genomic DNA was extracted according to Afanador et al. (1993). Four pairs of primers were designed to amplify the exon regions of the candidate gene in these six lines. Primer design was based on sequence alignment with G19833 reference genome version 1.0 (Schmutz et al., 2014) and subsequently updated for version 2.1. PCR reaction volume was 25 μL containing 30 ng of genomic DNA, 1X Taq buffer, 1.8 mM of MgCl_2_, 0.4 mM of dNTPs mix (Promega^®^), 0.25 μM of each primer (forward and reverse), and 1 U of homemade *Taq*1 polymerase under the following amplification conditions: heating for 2 min at 95°C, followed by 40 cycles at 94°C for 20 s, 57°C for 30 s and 72°C for 90 s, and a final extension at 72°C for 5 min. All the PCR amplifications were performed in a PCR Eppendorf Mastercycler (Eppendorf AG, Hamburg, Germany). PCR fragments were visualized by gel electrophoresis on 2% (w/v) agarose. Sanger sequencing of the PCR fragments was performed by Macrogen^®^ (Seoul, South Korea). Geneious 9.1.2^4^ was used for sequence trimming, alignment, and polymorphism discovery (Kearse et al., 2012).

Candidate gene SNPs between the six *bgm-1* lines and four reference genome accessions (=*Bgm-1*) were selected for marker development. Primers were designed to amplify the SNP mutations using the same protocols for the T_*m*_ shift assay described above. Candidate gene markers were evaluated for co-segregation with BGYMV reaction across ∼1,000 lines (described in detail below) from a RIL population, breeding populations, diversity panels, wild accessions, and well-known and original source resistance lines. Alternatively, the resulting PCR products were evaluated for polymorphisms on 4% agarose gels in 1 × Tris-Borate-EDTA buffer on a Bio-Rad^®^ (Sub-cell Model 192, Hercules, CA) gel electrophoresis system during three h at 100 volts. GelRed^®^ (Biotium^®^) was added to the gel for visualization of the DNA bands on a UVP GelDoc-It^®^ 310 Imaging system (UVP, Cambridge, United Kingdom).

Independently GWAS in the CIAT breeding population and BASE diversity panels (described below) was used to target a candidate gene for *bgm-1*. An annotated gene consistently occupying the most significant peak position in the GWAS results for the different populations was identified. A SNP within the gene was converted to a STARP (semi-thermal asymmetric reverse polymorphism) PCR marker to facilitate subsequent assays and evaluate MAS for the candidate gene. Five primers, including two universal primers and three SNP-specific primers, were developed for the STARP PCR. 40 ng for each 20 μl PCR reaction were prepared and programmed as described by Long et al. (2017). The SNP alleles were detected based on the size separation on 3% TBE agarose gel with 125V for 1.5 hrs. The STARP marker was also converted to a T_*m*_-shift assay marker as described above.

### RIL Populations for Identifying QTL Conditioning BGYMV Resistance

Two RIL populations were analyzed for QTL conditioning resistance to BGYMV. The DOR 364/XAN 176 (DX) population of 79 F_5:7_ RILs originally identified the BGY4.1 and BGY7.1 QTL using a selective mapping approach (Miklas et al., 1996). XAN 176 is susceptible to the BGYMV pathogen. DOR 364 expresses a quantitative resistance exhibited by moderate chlorosis and yield retention despite severe pathogen pressure and carries the *Bgm-1* allele. DOR 364 was derived from combining different sources “Porrillo Sintetico,” BAT 1215, and “Turrialba,” each possessing intermediate levels of quantitative resistance to BGYMV (Beebe and Corrales, 1991). The DX RILs were rated for reaction to BGYMV under natural infection in the field by Miklas et al. (1996) on a 1–9 scale for leaf chlorosis. The detected QTL, BGY4.1 and BGY7.1, were both consistently associated with reduced chlorosis across three field trials and had similar PVE (phenotypic variance explained). Together the QTL had an additive effect of 60% PVE for the Spring planted field trial. Miklas et al. (2000) generated a genetic linkage map for the same DX population with 156 mostly RAPD (random amplified polymorphic DNA) markers, but this map only slightly improved upon the original genetic intervals for BGY4.1 and BGY7.1. Eventually, the W12_700_ RAPD marker nearest the BGY4.1 peak was converted to a SCAR SW12_700_ (Singh et al., 2000) which facilitated MAS of the QTL. For this study, we re-analyzed these QTL in the DX population by using the same leaf chlorosis data from Miklas et al. (1996) but with a denser linkage map populated with SNPs generated by the BARCBean6K_3 BeadChip assay (Song et al., 2015). Leaf tissue was collected from an individual plant for each RIL and parents grown in the USDA-ARS greenhouses at Prosser, WA, United States. Genomic DNA was isolated from 20 mg of leaf tissue using a Qiagen DNeasy 96 Plant Kit (Hilden, Germany) and sent to the USDA-ARS, Soybean Genetics and Improvement Laboratory, Beltsville, MD for the BARCBean6K_3 BeadChip assay. The SNP genotyping was conducted on the Illumina platform by following the Infinium HD Assay Ultra protocol (Illumina Inc., San Diego, CA) (Song et al., 2015). The SNPs positioned were updated by alignment to the v2.1 reference genome assembly of G19833. Markers with > 20% missing data or significant deviation from the expected Mendelian segregation ratios as determined by chi-square analysis were removed. Further filtering (e.g., redundant markers) was performed during linkage map construction using MapDisto (details below).

The DOR 476/SEL 1309 (DS) population of 100 F_5:7_ RILs from CIAT was used by Blair et al. (2007) to locate *bgm-1* on Pv03 of the core genetic linkage map and to develop and characterize SCAR markers SR2 and SR21 developed from the original codominant R2_570/530_ RAPD marker (Urrea et al., 1996). The BGYMV resistance of DOR 476 derives from a combination of sources in its pedigree: A429, which combines resistances from Garrapato (*bgm-1*) and Porrillo Sintetico, and DOR 364 (described above). Blair et al. (2007) generated phenotypic disease reaction data for the DS RILs by mechanical inoculation in the greenhouse with a BGYMV strain from Guatemala. For this study, field reaction for the DS RILs, rated from 1 to 9 based on chlorosis and overall general performance in a field trial with two 4 m single-row replications at the San Andres Experiment Station of CENTA (the National Center for Agricultural Technology) in El Salvador in 2012, naturally infected with BGYMV, was used for QTL mapping and haplotype analysis of candidate gene markers for *bgm-1*. BGYMV is endemic in the region, and disease severity is especially intense in December plantings under irrigation that follow the commercial production season when inoculum has increased and when the dry season has set in, permitting large and reliable *Bemisia* whitefly vector populations. In this season, it is unnecessary to plant spreader rows before the nursery to increase inoculum, although a susceptible local check is planted to monitor disease build-up. Susceptible variety “Rojo de Seda” routinely registers disease symptoms of 7 or 8, while resistant varieties present symptoms in the range of 2.5–3.5.

Although 84 mostly SSR markers (Pedraza et al., 1997; Blair et al., 2007) were previously assayed in the DS population, no QTL conditioning BGYMV resistance had ever been reported. We similarly extracted DNA and re-genotyped the DS population with 5298 SNPs from the BARCBean6K_3 BeadChip assay. The v2.1 positions (see above) of the SNPs were used for mapping. Filtered SNPs were used to develop a dense linkage map for QTL analysis of field reaction (1–9) to BGYMV.

### Linkage Map Construction and QTL Analysis

Genetic linkage maps for DX and DS populations were created using MapDisto version 1.8.1 (Lorieux, 2012) with an r_*max*_ of 0.24, LOD_*min*_ of 3, and the Kosambi function. The “order” and “ripple” functions for each sequence were used to optimize the order of each marker. Linkage groups were assigned to a physical common bean chromosome (v2.1) by using BLASTN results from Phytozome 12^5^. The map was visualized using MapChart (v. 2.30, Wageningen University and Research, Wageningen, Netherlands).

QTL analyses were conducted using R/qtl v1.39-5 (Broman et al., 2003) in R v3.3.0 (R Core Team, 2013). One-dimensional, single-QTL genome scans were performed using multiple imputations with a scanning interval of 1 cM (imputations = 1,000; error probability = 0.001). Two-dimensional genome scans were performed using Haley-Knott regression with the thresholds based on the results of 1,000 permutations at a 5% significance level to enable assessment of evidence for multi-QTL models involving additive or interacting loci.

### GBS and GWAS in BASE Diversity Panels and CIAT Breeding Population

DNA from 412 CIAT breeding lines was extracted using MagMAX^TM^ Plant DNA Isolation Kit. The GBS libraries were prepared using the two-enzyme (MseI and Taqα1), low-pass sequencing SNP set protocol (Schröder et al., 2016). The libraries were sequenced at HudsonAlpha Institute for Biotechnology using an Illumina HiSeq sequencer. Fastx barcode splitter and Fastx trimmer (Gordon and Hannon, 2010) were used to split and trim the barcodes from the sequences. A quality threshold of 20 and a minimum trimmed length of 80 bp were applied using sickle for a quality control before mapping. Samtools (Li et al., 2009) and BWA-MEM (Li, 2013) mapping programs were used to index, sort, and align the data against the G19833 reference genome assembly. SNPs were called using GATK UnifiedGenotyper (v3.3) with a minimum confidence threshold of 30 (McKenna et al., 2010). 168,477 SNPs were identified among the 412 breeding lines. After filtering for MAF 0.05, 114,163 SNPs were used in GWAS analysis. For the BASE_120 and BASE_MESO populations, among the 211,7764 SNPs reported among a larger Middle American gene pool population (Oladzad et al., 2019), 125,745 SNPs polymorphic in the two populations, with a MAF > 0.05, were used for the GWAS. Imputed and unimputed SNP data sets can be found at: http://arsftfbean.uprm.edu/beancap/research/.

The CIAT dry bean breeding population for Central America representing 412 lines from 38 different pedigrees, each with at least one parent possessing *bgm-1* (based on markers and disease reaction), were phenotyped for reaction to BGYMV in the field in El Salvador in 2015 in the same conditions as described for the DS population above. Disease reaction, based primarily on severity of leaf chlorosis, was rated from 1 to 9 at mid pod fill.

The BASE 120 panel, with 93 lines of Middle American origin, 22 lines of Andean origin, and four tepary beans (*Phaseolus acutifolius*), was phenotyped for BGYMV reaction in El Zamorano, Honduras, in October 2014 based on disease incidence as a percentage of plants infected (exhibiting chlorotic symptoms). Lines were planted in single-row plots of 3 m row length. Rows were spaced 0.6 m apart. There were three replications. This trial experienced a naturally occurring BGYMV infection that was moderately severe but not uniform across the trial. Thus, for GWAS, the highest disease incidence among all replications represented the BGYMV reaction for each line. A second BASE_Meso panel with 119 lines (56 lines in common with the BASE_120) mostly of race Mesoamerican origin and suited for production in Central America was planted in El Zamorano, Honduras, in December 2015, for seed increase and observation. This trial experienced severe BGYMV infection. Each line was rated at late pod-fill growth stage as resistant or susceptible based on absence versus presence of chlorosis symptoms, respectfully. A few resistant lines with deformed pods were noted.

GWAS analyses for all panels and populations were performed using GAPIT R package (Lipka et al., 2012; R Core Team, 2013) for score data (1–9) and genABLE R package (Aulchenko et al., 2007) for binary data. For each GWAS analysis, principal component analysis (PCA matrix) as a fixed effect and relatedness (kinship matrix) as random effects were tested in general linear and mixed linear models. 2PCA for CIAT breeding lines and 3PCA for BASE_120 and BASE_MESO, explaining 25–50% of the variation in each population, obtained from the Prcom function in R, and the kinship matrix computed from EMMA algorithm implemented in GAPIT, were used in the related GWAS analyses. Finally, the best GWAS model was obtained from MSD value (Mamidi et al., 2011). SNPs with MAF > 0.05 in each population was used to generate Manhattan and QQ plot in R package gap (Zhao, 2007). The annotated genes discovered in the most recent common bean reference genome were used to search for the candidate genes within a 50 kb interval of the SNP peaks.

## Results

### NAC Candidate Gene for *bgm-1*

The NGSEP pipeline identified 45 SNPs between the *bgm-1* and *Bgm-1* groups of resequenced lines, and primers were developed for T_*m*_-shift assay for 14 of these SNPs (Supplementary Table S1A). The haplotypes for the 14 SNP markers, spanning 2,265,754–2,906,614 bp on chromosome Pv03, were compared with BGYMV reaction in the DS RIL population, which segregates for *bgm-1* (Supplementary Table S1B). Informative SNP haplotypes in the DS population narrowed the *bgm-1* interval to 2,578,215–2,777,244 bp on Pv03. Within this interval, there are 13 gene models. Among these gene models, one gene model, *Phvul.003G027100*, was annotated as a NAC domain transcriptional regulator superfamily protein, a homolog of a geminivirus replication factor in tomato (Selth et al., 2005).

**FIGURE 1:**
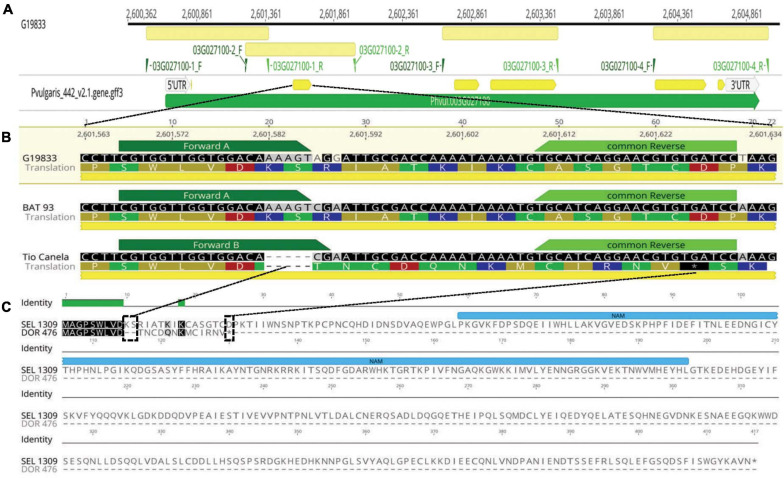
Candidate *Phvul.003G027100* NAC gene model for *bgm-1* in *Phaseolus vulgaris:* **(A)** Four primer pairs designed for amplifying the six exons of candidate gene *Phvul.003G027100* (Supplementary Table 1B). Physical positions from reference genome v2.1 (G19833). **(B)** T_*m*_-shift-assay marker (PvNAC1) designed for amplification of 5 bp indel region in second exon of *Phvul.003G027100* in accessions G19833 (*Bgm-1*), BAT 93 (*Bgm-1*) and Tio Canela 75 (*bgm-1*). **(C)** Protein alignment of *Phvul.003G027100* candidate gene for *bgm-1* (DOR 476) revealed a stop-gain mutation in position 23. Effect of the indel frameshift mutation on transcription of the candidate gene among ten lines—three with and seven without *bgm-1* modeled by Geneious^®^ 9.1.8 software.

**FIGURE 2:**
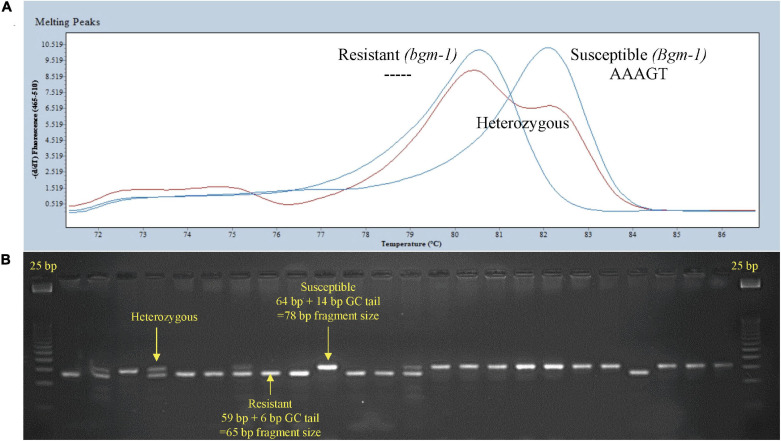
The CB_475_v3 marker developed for the five bp indel region in exon 2 of the candidate. **(A)** Phvul.003G027100 NAC gene for bgm-1 visualized by melting curve Tm-shift assay and **(B)** by gel electrophoresis.

Targeted sequencing for the ORF of *Phvul.003G027100* comprising six exon regions (Figure 1A and Supplementary Table 2) revealed seven SNPs and one polymorphic indel within the gene. The indel Pv03: 2,601,582–586 located in the second exon co-segregated with BGYMV phenotype among the nine resequenced lines (Table 1). The five bp indel (deletion) (Figure 1B) is predicted to cause a frameshift mutation, starting with AA 10 that results in a stop codon at codon 23, which is likely to disrupt transcription of the NAC gene (Figure 1C). A T_*m*_ shift assay marker named PvNAC1 was developed for detection of the indel, and the primers are described in Table 2. With the PvNAC1 marker, *bgm-1* resistant lines (with the five bp deletion) gave a lower temperature peak (T_*m*_ ∼80°C) compared to the *Bgm-1* susceptible lines (T_*m*_ ∼82.5°C) (Figure 2A). The PCR product could also be scored on a 4% agarose gel as an indel with 13 bp deletion (5 bp from NAC indel plus eight bp from the difference between GC tails of each forward primer). The susceptible line exhibited a fragment of 78 bp, including a 14-bp GC tail; whereas, the resistant line exhibited a fragment of 65 bp, including a 6-bp GC tail (Figure 2B).

**TABLE 1:** Polymorphisms detected within the exon regions for NAC candidate gene *Phvul.003G027100* among nine genotypes—three with and six without BGYMV resistance conditioned by *bgm-1.*

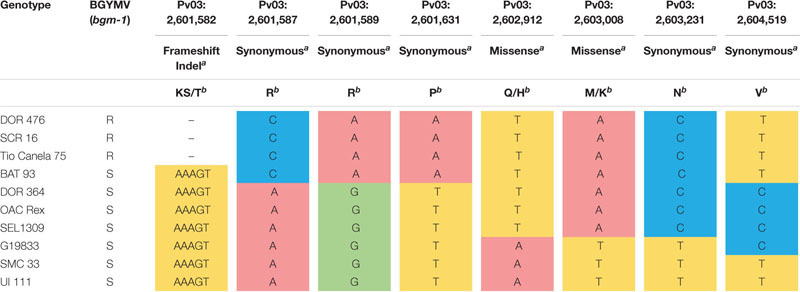

*^*a*^Variant type.^*b*^Amino-acid substitutions in protein-coding regions.*

**TABLE 2:** Primers used to generate markers for *bgm-1* and QTL conditioning resistance to BGYMV.

**T_*m*_ shift assay markers**	**Gene/QTL**	**Position**	**REF**	**ALT**	**Gene model**	**Annotation**
PvNAC1	*bgm-1*	2,601,581	AAAAGTA, AAAAGTC	–	Phvul.003G027100	NAC (No Apical Meristem) domain transcriptional regulator superfamily protein
Primers Fa_gcgggcagggcggcCGTGGTTGGTGGACAAAAGT; Rev_GGATCACACGTTCCTGATGCA; Fb_gcgggcCTCGTTCATCAACCTTGG						
S03_2524224	CHUP1	2,524,224	G	C	Phvul.003G026100	Hydroxyproline-rich glycoprotein family protein
Primers Fa_gcgggcagggcggcCTCGTTCATCAACCTTGC; Rev_CTTTATCCAGCCACCACAGAAT; Fb_gcgggcCTCGTTCATCAACCTTGG						
S04_2531038	BGY4.1	2,531,038	G	A	Phvul.004G022000	cytochrome P450, family 82, subfamily C, polypeptide 4
Primers Fa_gcgggcagggcggcCCAAAGGTTTCAGTCATGTC; Rev_GGCAGAAGAATGTGTCCAGG; Fb_gcgggcTCCAAAGGTTTCAGTCATGTT						
S1137_407	BGY7.1	Scaf_1137 _407	CCT	–	Phvul.L001619.1	Putative Mediator of RNA polymerase II transcription subunit 18
Primers Fa_gcgggcagggcggcGCTGGAGTTACTCCCTcct; Rev_TAATCCTCCTTAAACGTGATGGT;? Fb_gcgggcTTGCTGGAGTTACTCCCTAG						
S08_9202267	BGY8.1	9,202,267	T	A	Phvul.008G091500	Pentatricopeptide repeat (PPR) superfamily protein
Primers Fa_ggcgggcagggcggcCAAGCATCAATGAAGCTCA; Rev_TCATTGGTCGCTTTAGTTTGAA; Fb_gcgggcCAAGCATCAATGAAGCTCT						
STARP marker for CHUP1		2,524,224	G	C	Phvul.003G026100	Protein CHUP1, Chloroplastic
Universal primers PEA-1-F_AGCTGGTT-Sp9-GCAACAGGAACCAGCTATGAC-3′; PEA-2-R_ACTGCTCAAGAGAGAGAG-Sp9-GACGCAAGTGAGCAGTATGAC-3′						
Specific Primers Fa_GCAACAGGAACCAGCTATGACCACAGAATAcTTATCATCTaATTTTATTCCTTcTc; Rev_GCTTTCCCTTCAGGCCAGTCAAAGT; Fb_GACGCAAGTGAG CAGTATGACCACAGAATAgTTATCATCTcATTTTATTCCTcATg						

### CHUP1 Candidate Gene for *bgm-1*

The GWAS analysis of CIAT’s 412 breeding lines detected a highly significant interval on Pv03: 2.43–2.54 Mbp with a peak SNP S03_2524224 [−log10(*P*-value) = 35.23] (Table 3 and Supplementary Table 3A). Searching for the candidate genes revealed that SNP S03_2524224 is located inside the gene model *Phvul.003G026100*, an ortholog of the Arabidopsis CHUP1 (Chloroplast Unusual Positioning) gene that contributes to the movement of chloroplasts on microfilaments in response to changes in light intensity, and upon silencing reduces virus infection (Angel et al., 2013). A STARP marker named PvCHUP1 was generated for the SNP S03_ 2524224 within the CHUP1 gene. The primers are described in Table 2. Primers were developed for T_*m*_ shift assay of the SNP S03_2524224 as well.

**TABLE 3:** Summary of the GWAS results for reaction to BGYMV in three populations highlighting the *bgm-1* region on chromosome Pv03.

**Population**	**Significant interval (Mbp)**	**Peak SNP position (bp)**	**log10 (p.value)**
CIAT lines	2.43–2.54	2,524,224	35.23
	2.58–2.64	2,635,286	31.52
	2.90–3.07	3,024,153	17.73
BASE_120	2.43–2.52	2,523,551	6.82
	2.63–2.64	2,636,541	5.57
	2.87–3.01	2,911,214	7.39
	3.04–3.10	3,065,589	7.93
BASE_MESO	1.99–2.03	2,029,979	4.85
	2.43–2.64	2,438,299	6.63
	2.87–3.01	2,919,423	5.01

### Comparison of NAC and CHUP1 Candidate Gene Markers

**FIGURE 3:**
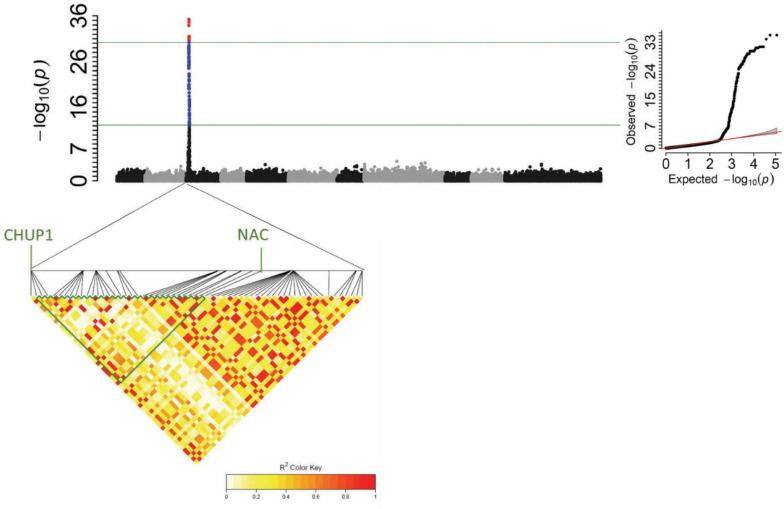
Manhattan and QQ plots from GWAS (Supplementary Table 3B) in 412 CIAT breeding lines evaluated (1–9 disease score) in El Salvador (2015). With an LD heat-map view of the significant genomic region from 2.52 to 2.63 Mbp for *bgm-1* on Pv03. The positions of candidate genes are shown.

**FIGURE 4:**
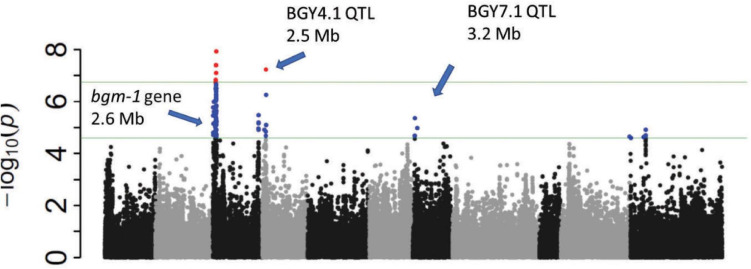
Manhattan plot from GWAS (Supplementary Table 3C) in BASE_120 evaluated for % infection in Honduras in 2014. Tepary and Andean beans were excluded.

**FIGURE 5:**
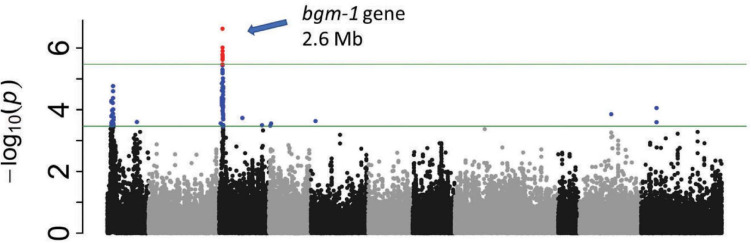
Manhattan plot from GWAS (Supplementary Table 3D) in BASE_Meso evaluated (R vs. S reactions) in Honduras in 2015.

The CIAT breeding lines were screened with the PvNAC1 marker, and the data was added to the GWAS SNP set, and the GWAS was again performed (Figure 3). The *P*-value for the CHUP1 S03_ 2524224 SNP was slightly more significant than the PvNAC1 marker (Supplementary Table 3). GWAS detected the CHUP1 region in BASE_120 panel, and CHUP1 and NAC gene regions associated with *bgm-1* resistance in the BASE_MESO population as well (Figures 4, 5). The genomic region 2.5–2.6 Mb spanning these genes is in high LD, resulting in nearly complete co-segregation of the CHUP1 and NAC markers for the 412 CIAT lines (Figure 3 and Supplementary Table 4A). Subsequently, the BASE_120 was assayed with PvCHUP1 and PvNAC1 markers, and eight recombinants were observed (Supplementary Table 4B). Seven race Durango accessions CO 46348, CO 91212-4, Croissant, GN9-4, Matterhorn, PK-7-4, and USRM-20 and one race Mesoamerican (Zorro) accession possessed the resistance allele for the PvCHUP1 marker and the susceptible allele for the PvNAC1 marker. Only three of these eight lines had resistant reactions. There were five additional accessions in the BASE_Meso population with the resistance allele for PvCHUP1 and susceptible allele for PvNAC1 and all were susceptible to BGYMV (Supplementary Table 4C). The survey of both markers was expanded to the race Durango Diversity Panel (DDP; ∼191 accessions), where the resistance allele for the PvCHUP1 marker was observed in 80% of the population (Supplementary Table 4D). Conversely, the resistance allele for the PvNAC1 marker was absent in all but one DDP accession PR0401_259 with *bgm-1* source from Garrapato in its pedigree and reported to have resistance to BGYMV (Beaver et al., 2012). PR0401_259 is mostly of race Mesoamerican origin but served well as a control for *bgm-1* in this instance. It is noteworthy that race Durango materials such as UI-114 and UI-36 exhibited resistance/tolerance to BGYMV in studies conducted by Morales and Niessen (1988) and Morales and Singh (1991). It would be worthwhile to revisit the value of those lines and the resistant race Durango lines CO 46348, PK7-4, and USRM-20 observed in this study in breeding for resistance to BGYMV. These comparative results thus far support the Phvul.003G027100 NAC gene as the better candidate for *bgm-1*.

### BGY4.1 and BGY7.1 QTL

**FIGURE 6:**
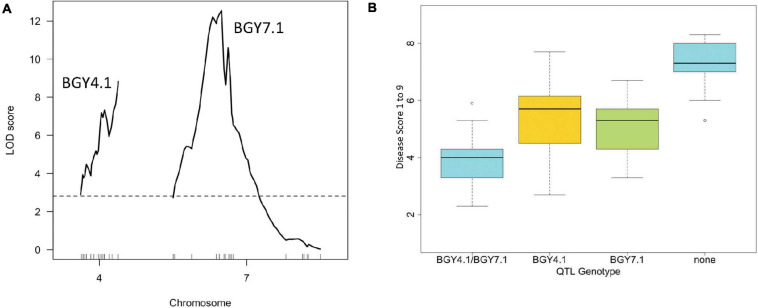
Genetic mapping for resistance to BGYMV (1–9) evaluated in the DOR 364 x XAN 176 (DX) recombinant inbred population in Puerto Rico in spring 1994 (Miklas et al., 1996): **(A)** LOD profiles obtained by two-dimensional genome scans. The BGY4.1 and BGY7.1 QTL models were fitted and refined using the multiple imputation method. Dashed line denotes significance at the 0.05 probability level and **(B)** interaction between BGY4.1 and BGY7.1 QTL for resistance to BGYMV (see also Supplementary Figure S1).

A linkage map (736 cM) for the DX RIL population (Supplementary Figure 1) was generated from 380 SNPs from the BARC_3 BeadChip assay and 2 SCAR markers [SW12 used previously to detect BGY4.1 and SAP6, which detects a QTL for common bacterial blight (*Xanthomonas axonopodis pv. phaseoli* [Smith] Vauterin et al.)] resistance. Subsequently, 15 additional SNPs and one indel designed from T_*m*_-shift assays were used to further saturate the BGY4.1 and BGY7.1 QTL intervals. These additional markers were identified from resequenced genotypes from Lobaton et al. (2018), the DDP (this study), and for DOR 364 and XAN 176 parental genotypes (GBS data) included in the Middle American Diversity Panel (MDP) (Moghaddam et al., 2016). Note that Pv04 and Pv08 were split into two linkage groups each, Pv04a—Pv04b and Pv08a—Pv08b, because they had gaps greater than 50 cM. With these adjustments, the increased map length and density of 1.9 cM was a significant improvement upon the 511 cM map length with 5.4 cM spacing between markers reported by Miklas et al. (2000). Moreover, the QTL analysis identified two QTL (Figure 6A), BGY4.1 on Pv04 and BGY7.1 on Pv07, previously identified (Miklas et al., 1996). BGY7.1 accounted for 40.6% phenotypic variation explained (PVE) while BGY4.1 exhibited 25.3% PVE (Table 4), and the two QTL acted additively (Figure 6B) as previously observed.

**TABLE 4:** QTL identified in the DX RIL population.

**Chr**	**QTL**	**Start (bp)**	**End (bp)**	**DF**	**SS**	**LOD**	***R*^2^ (%)**	**Probability (F)**	**Additive effect**
Pv04	BGY4.1	2,318,252	2,855,147	2	53.73	8.8	25.3	4.24E-09***	0.83
Pv07	BGY7.1	2,706,840	3,525,083	2	86.08	12.5	40.6	1.25E-12***	1.03

***p0.001.

BGY4.1 mapped to a relatively narrow genomic interval, 2,318,252–2,855,147 Mb (Table 4). Adding four additional SNP markers and subsequent haplotyping further narrowed the BGY4.1 interval to 2,356,775–2,656,383 bases (Supplementary Table 5). Thirty-three genes were found within this interval, including an eight-member cytochrome P450 gene cluster (Supplementary Table 6). A similar cytochrome P450 protein family in *A. thaliana* was upregulated following geminivirus AC2/C2 protein infection (Babu et al., 2018). Haplotyping in the DX population (Supplementary Table 5) revealed the S04_2531038 SNP marker (missense variant) located within the second exon of the *Phvul.004G022000* CYP82C4 gene as having the best potential for MAS of BGY4.1. Primers for Tm-shift assay of S04_2531038 are described in Table 2.

The BGY7.1 QTL in DX population mapped to a 2,724,611 to 3,525,083 bp interval at the proximal end of Pv07 (Table 4, Supplementary Table 5, and Figure 6). This position for BGY7.1 overlaps the dominant *Bct-1* gene (Larsen and Miklas, 2004), identified in our laboratory by a tightly linked SNP marker S07_2,970,381 (Soler-Garzón et al., 2018). *Bct-1* conditions resistance to *Beet curly top virus* (BCTV), a Curtovirus, and co-segregates with a major QTL conferring resistance to *Bean dwarf mosaic virus* (BDMV) (Miklas et al., 2009), a Begomovirus. Soler-Garzón et al. (2018) identified *Phvul.007G036300* (2,969,877–2,971,300) as a candidate gene model for *Bct-1*. The gene has probable exonuclease activity (Pfam domain PF09810 EXO5; exonuclease V), which has been reported to play a role in the cleavage of viral DNA from bacteriophages (Hanley-Bowdoin et al., 2013). Sequencing *Phvul.007G036300* from the DOR 364 and XAN 176 parents did not reveal any polymorphisms. But we used the WGS data for the DDP to identify two SNPs bordering *Phvul.007G036300*, which mapped in DX (Supplementary Tables 5,7). SNP S07_2966197 is a missense variant in *Phvul.007G036200*, a putative transmembrane protein gene. SNP S1137_407 is associated with an indel (at base 407) in a scaffold (S1137) mapped in UI-111 reference genome from 3,116,364 to 3,117,307 bases on Pv07. The scaffold 1137 is positioned between ∼ 2,977,925 to 3,001,104 bases in v2.1 G19833 reference genome. Both SNPs were converted to T_*m*_-shift assays (Table 2). These SNPs were also assayed in the other populations/panels (Supplementary Tables 4A–D), where SNP S07_2966197 was most polymorphic in Durango and S1137_407 in Mesoamerican backgrounds.

GWAS in BASE_120 revealed significant regions on Pv04 and Pv07 in addition to *bgm-1* (Figure 4). The four tepary and 22 lines of Andean origin were excluded from this analysis. The Pv04 interval from 1.33 to 2.52 Mb in BASE_120 overlaps the 2.47–2.55 Mb genomic interval for the BGY4.1 QTL in the DX RIL population. The peak SNP (2.51 Mb) from GWAS is within the cluster of eight cytochrome P450, family 82, subfamily C, polypeptides. The less significant region from 2.55 to 5.16 Mb on Pv07 overlaps the 2.72–3.52 Mb genomic interval for the BGY7.1 QTL in the DX RIL population. Although the 3.21 Mb GWAS peak is outside this narrow interval in DX, we assume for now that the same BGY7.1 QTL is present in both populations.

### BGY8.1 QTL

**FIGURE 7:**
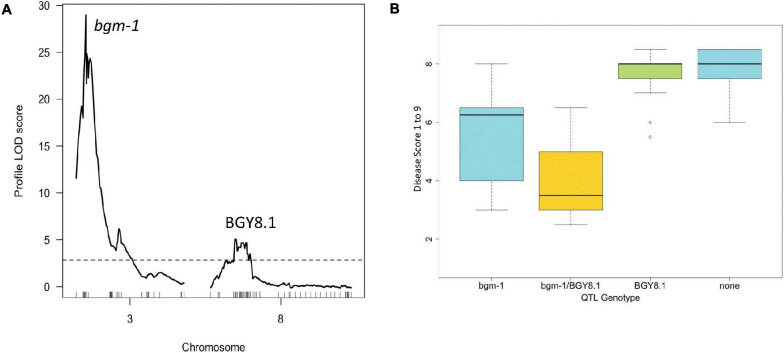
Genetic mapping for resistance to BGYMV (1–9) evaluated in El Salvador in 2012 in DOR 476/SE. (DS) recombinant inbred population: **(A)** LOD profiles obtained by one-dimensional and two-dimensional genome scans. QTL models were fitted and refined using the multiple imputation method. Dashed line denotes significance at the 0.05 probability level and **(B)** interaction between *bgm-1* and BGY8.1 for resistance to BGYMV.

575 SNPs, the original SR2 SCAR, the new PvCHUP1 and PvNAC1 markers, and six SNPs from resequencing data (Lobaton et al., 2018) were used to generate a linkage map for the DS RIL population. The map had an average marker spacing of 1.6 cM and an overall map length of 916.7 cM (Supplementary Figure 2). Note that Pv07 was split into two linkage groups Pv07a and Pv07b. The QTL analysis for the disease severity scores from a 2012 evaluation in El Salvador detected the recessive *bgm-1* resistance gene on Pv03 with major effect (75% PVE) and a minor QTL (7% PVE) on Pv08 with epistatic effect (Figures 7A,B). This novel QTL named BGY8.1 has no effect on its own but exhibits a major effect when combined with *bgm-1* gene. A similar epistatic interaction is apparent for *Bgp-1* (reduced pod deformation) which is only observed in the presence of *bgm-1* (Acevedo-Román et al., 2004). A wide 6.2–13.6 Mb interval was observed for BGY8.1, and the peak was 9.20 Mb. Interestingly there was one recombinant line (DS42) in the DS population with the resistance allele for PvCHUP1 marker and susceptible allele for PvNAC1 marker (Supplementary Table 1B). This recombination equates to 0.53 cM between the genes, which fits the expected recombination rate for the 80.6 Kb distance between them. This recombinant line (DS42) had an intermediate resistance score of 4.0, whereas the DS lines with both resistance-linked alleles had an average score of 4.7.

**FIGURE 8:**
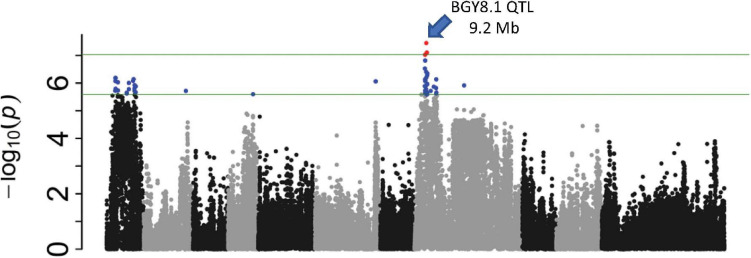
Manhattan plot for a subpopulation of 128 lines from the CIAT population with *bgm-1* (PvNAC1 gene marker) used for detection of QTL (BGY8.1) needing *bgm-1* for expression.

**FIGURE 9:**
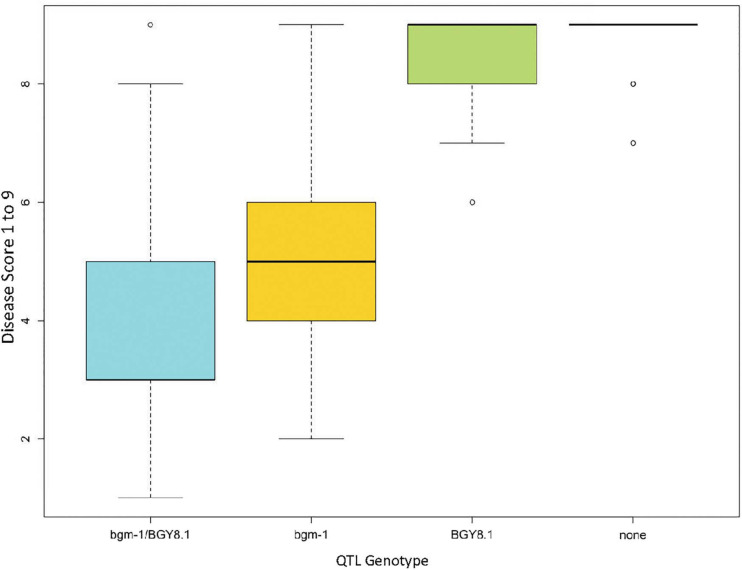
Boxplot showing interaction between *bgm-1* (PvNAC1 marker) and BGY8.1 QTL (SNP S08_9202267) in the CIAT population of 412 lines.

A separate GWAS for the CIAT population conducted with a subset of 128 lines (15CF6) with *bgm-1* (possessing the resistance allele for PvNAC1 marker) clearly showed a significant interval from 8.7 to 9.2 Mb on Pv08 (Figure 8 and Supplementary Table 3E). The 9.0 Mb peak position is within the genomic interval and near the peak position (9.2 Mb) for the BGY8.1 QTL observed in the DS RIL population. For this 9.2 region on Pv08, we identified one SNP S08_9202267 from the resequencing data for 40 lines (Lobaton et al., 2018) which segregated in the DS RIL and CIAT breeding populations and adapted it for T_*m*_-shift assay to use for detection of the BGY8.1 QTL. SNP S08_9202267 is a missense variant within the third exon of the *Phvul.008G091500* pentatricopeptide repeat (PPR) superfamily protein gene. However, we do not consider *Phvul.008G091500* to be a candidate gene for the QTL. The SNP S08_9202267 marker was then used to measure the effect of the QTL on disease severity score for all 412 CIAT lines. The results clearly show the same epistatic interaction as observed in the DS RIL population, that the QTL exhibits little to no effect in the absence of *bgm-1*, but it has a significant effect for reducing disease severity in the presence of *bgm-1* (Figure 9). Thus, GWAS with the CIAT advanced breeding lines validated the BGY8.1 QTL and its epistatic interaction with *bgm-1* as observed in the DS RIL population.

The effects of the above markers linked with *bgm-1* (PvNAC1), BGY4.1 (S04_2531038), BGY7.1 (S1137_407), and BGY8.1 (S08_9202267) on the mean level of reaction to BGYMV were examined in the CIAT and BASE_120 populations (Table 5 and Supplementary Tables 4A,B). For the CIAT population, lines with both the *bgm-1* and BGY8.1 resistance allele linked markers, on average, were the most resistant. CIAT breeding lines with the resistance allele for BGY4.1 marker were more susceptible, indicating MAS for this QTL-linked marker had a negative influence on resistance in that population. The BGY7.1 linked marker had no apparent influence on reaction to BGYMV in the CIAT population and thus was omitted from Table 5. Conversely, the BGY4.1 and BGY7.1 linked markers had a positive association with resistance in the BASE_120 population. In fact, lines with both BGY4.1 and BGY7.1 resistance allele linked markers, on average, were as resistant as the lines with the *bgm-1* linked marker only. Clearly, resistance was enhanced when *bgm-1* was combined with BGY4.1 or BGY7.1. The epistatic effect whereby BGY8.1 in combination with *bgm-1* improved resistance as seen in the DS and CIAT populations was not evident in the BASE_120 population.

**TABLE 5:** Mean effect of *bgm-1* (PvNAC1) and QTL-linked markers for BGY4.1 (S04_2531038), BGY7.1 (1143_407)^*a*^, and BGY8.1 (S08_9202267) on disease reaction in two populations.

**Genes/QTL**	**Lines**	**Disease**	**StdDev**
CIAT population	no.	1–9 score	
*bgm-1*/BGY8.1	54	3.5	1.3
*bgm-1*	64	4.2	1.3
*bgm-1*/BGY4.1	38	5.1	1.4
*bgm1*/ BGY4.1/BGY8.1	13	5.2	2.2
BGY8.1	58	8.4	0.7
BGY4.1/BGY8.1	24	8.6	0.5
BGY4.1	59	8.8	0.4
None	67	8.8	0.5
Missing data	35	7.4	1.9
Grand total	412	6.8	2.4
BASE_120 population		% severity (high score)	
*bgm-1*/BGY4.1/BGY7.1	3	7.3	4.6
*bgm-1*/BGY7.1	8	16.9	9.6
*bgm-1*/BGY7.1/BGY8.1	6	20	15.5
BGY4.1/BGY8.1	5	25.4	32.4
BGY4.1/BGY7.1	5	27	18.6
*bgm-1*	3	36.7	20.8
BGY4.1/BGY7.1/BGY8.1	3	36.7	20.8
BGY7.1	19	42.1	19.9
*bgm-1*/BGY8.1	7	43.1	31
BGY4.1	1	50	
BGY8.1	36	54.6	26.1
BGY7.1/BGY8.1	12	66.7	21
None	6	78.3	13.3
Missing data	6	40	26.1
Grand total	120	44.9	27.4

*^*a*^BGY7.1 marker was omitted from the CIAT population because of neutral effect.*

## Discussion

The recessive resistance gene *bgm-1* is the most effective and widely deployed gene to control BGYMV. We sought to improve MAS and identify the candidate gene for *bgm-1*. A stepwise approach comparing resequenced lines with and without the gene and haplotyping in a bi-parental population (DS RILs) segregating for the gene was used to narrow the genomic interval for *bgm-1*. A putative candidate within the interval, *Phvul.003G027100*, which encodes a NAC domain transcription factor protein, was sequenced across four genotypes (DOR 476, SCR 9, SCR 16, and Tio Canela 75) with and five (CAL 96, SCR 2, SEN 56, SEA 5 and SMC 33) without *bgm-1*. Among the exons, a five bp deletion in the second exon, co-segregated with resistance across the resistant genotypes. A combined real-time T_*m*_-shift assay and agarose gel-based marker (PvNAC1) was developed to detect this polymorphism. Separately, GWAS detected a CHUP1 homolog (*Phvul.003G026100*), which is in high LD with the NAC gene, as a putative candidate gene for *bgm-1*. The inclusion body protein P6 of *Cauliflower mosaic virus* (CaMV) interacts with CHUP1 to facilitate intercellular movement of the virus (Angel et al., 2013; Schoelz et al., 2016). According to Oikawa et al. (2003, 2008), the mutant chup1 prevents chloroplast transport, and according to Angel et al. (2013), silencing CHUP1 reduced the ability of the virus to infect host plants. However, expanded assays in this study revealed the PvCHUP1 SNP marker occurred in many more susceptible materials than the PvNAC1 marker; therefore, CHUP1 was not pursued in-depth as a candidate gene for *bgm-1.*

Further evidence supporting the five bp deletion in the second exon of the NAC gene as the causative event that generated *bgm-1* is provided by the origin of the mutation itself. The CIAT breeding line A429 (Garrapato//Porrillo Sintetico/G 2115) was found to possess the highest level of resistance (low chlorosis, low stunting, low flower abortion) to BGYMV at the time (Morales and Niessen, 1988). Evaluation of the parents of A429 by mechanical inoculation in the glasshouse revealed all of them to be susceptible. Later studies (Velez et al., 1998) confirmed the low chlorosis reaction in A429 was conditioned by a recessive resistance gene *bgm-1* derived from Garrapato (G 2402), a landrace pinto from Mexico. The five bp deletion traces back to Garrapato, while susceptibility is associated with the wild-type allele. We observed that Garrapato was heterogeneous for the five bp deletion. Garrapato is of Durango Race origin, so we assayed 191 accessions for PvNAC1 marker in the Durango Diversity Panel (DDP), and none possessed the five bp deletion, except PR0401_259, a Mesoamerican line from University of Puerto Rico with *bgm-1* that traces back to Garrapato through its pedigree. Furthermore, the five bp deletion allele for the PvNAC1 marker, observed in more than 200 of 1,000 lines assayed, all trace back through their pedigrees to Garrapato via the CIAT breeding line A429. Lastly, wild *P. vulgaris* pooled DNA samples (Schmutz et al., 2014; Lobaton et al., 2018) and wild *P. vulgaris* single accessions (Blair et al., 2012) (representing more than 300 wild accessions) assayed for PvNAC1 lacked the five bp deletion, supporting this mutation as a post-domestication event.

The NAC domain genes represent a large family of transcription factors in plants, including three transcription factors: NAM, ATAF, and CUC2, which each possess a DNA-binding domain. In Arabidopsis, there are more than 100 NAC genes. The few NAC genes studied to date have exhibited diverse functions (Olsen et al., 2005), with some involved in plant defense against geminiviruses. The most relevant to our finding is the SlNAC1 NAC gene in tomato (*Solanum lycopersicum*) which supports *Tomato leaf curl virus* (a Begomovirus) replication (Selth et al., 2005). SINAC1 is upregulated and interacts with geminivirus enhancer proteins (REn) in infected yeast cells. Overexpression of SlNAC1 resulted in increased TLCV DNA accumulation. Anbinder et al. (2009) mapped a major effect QTL (Ty-5) conditioning resistant to TLCV to the SlNAC1 locus on chromosome 4 in tomato. The same populations segregated for four minor-effect QTL, which made it difficult to discern recessive inheritance for the QTL. A later study (Hutton et al., 2012) clearly showed a recessive allele *ty-5* at the SlNAC1 locus on chromosome 4 conditioned resistance to TLCV in the tomato cultivar “Tyking.”

The recessive inheritance for *bgm-1* fits a similar disruptive host NAC gene model conferring geminivirus resistance in common bean. The five bp deletion in the ORF at base 29 in the second exon for Phvul.003G027100 gene is predicted to cause a frameshift-stop mutation at base 67, disrupting generation of the NAC protein. This resistance model suggests absence of the functional NAC protein reduces viral replication and contributes to host resistance. The NAC role in defense has been well studied (Huang et al., 2017). The N-terminal region of NAC protein domains is highly conserved, whereas the C-terminal region is diverse. The protein for the *Phvul.003G027100* NAC gene candidate for *bgm-1* was blasted in NCBI, and the most significant hit (82% identity) was to a NAC domain gene (*Glyma.01G088200*) in *Glycine max*. The *Phvul.003G027100* protein alignment with SlNAC1 (*Solyc04g009440*) associated with geminivirus resistance in tomato revealed only 11.75% similarity, which is low, but *Phvul.003G027100* is orthologous with AT1G25580 (51.67% of similarity) and another SlNAC *Solyc05g009840* (60.24 %) in tomato. In *Oryza sativa*, 68% of OsNAC genes were differentially regulated in response to viral infection (Nakashima et al., 2007; Nuruzzaman et al., 2015). Given the likely involvement of *Phvul.003G027100* in resistance to BGYMV, we blasted its protein sequence in *Phaseolus vulgaris* v2.1. A total of 90 NAC domain/related genes were identified with the highest identity (58%) with *Phvul.002G328700* (Supplementary Figure 3). There were some NAC genes in the vicinity of BGY4.1, but not with BGY7.1 or BGY8.1 QTL. We blasted SlNAC1 in *Phaseolus vulgaris* 2.1 and the NAC genes most similar were on Pv09. In summary, to date, no other NAC related genes in *P. vulgaris* v2.1 other than the *Phvul.003G027100* candidate for *bgm-1* appears to be related to geminivirus resistance.

In the CIAT breeding population and two BASE populations, there were a few crossover reactions where resistant lines lacked, and susceptible lines possessed the NAC 5 bp deletion marker allele associated with *bgm-1*. The former could be attributed to likely presence of *bgm-2* (e.g., Badillo), other resistance sources, or disease escape due to early maturity (CELRK), and the latter to heterozygosity, seed mixes, or lack of additional resistance genes/QTL. One or two susceptible contaminant plants in an otherwise resistant line could result in a susceptible rating. The *bgm-1* gene is most effective when combined with other resistance factors. The recommended breeding strategy is MAS for *bgm-1*, followed by phenotypic selection for resistant reactions in the field. The PvNAC1 marker is clearly an improvement for MAS over previous *bgm-1* linked markers. It replaces the adjacent SNP S03_2601587 (CB_434) (Supplementary Table 4) and SNP S03_2643523 (CB_352) markers used temporarily by CIAT for MAS (Soler-Garzón et al., 2017), which were observed in susceptible accessions, none of which possessed a *bgm-1* parent in their pedigrees. Similarly, the original SR2 SCAR marker (Supplementary Table 4) was observed in some susceptible lines in the BASE_120 and BASE_Meso populations.

There were three race Durango lines (CO 46348, PK7-4, and USRM-20) in the BASE_120 trial which lacked *bgm-1* but exhibited high levels of resistance to BGYMV in Honduras. This is an interesting result because moderate resistance in some race Durango materials was previously observed. USRM-20 red bean from the USDA-ARS, Prosser WA breeding program, recently released as “Atillos” in Nicaragua, was reported to have tolerance to BGYMV by the Federación de Cooperativas para el Desarrollo. Morales and Niessen (1988) included race Durango cultivars NW-59, NW-63, and NW-395 in their survey for resistance sources because they were bred for resistance to BCTV. The same study tested the *Bean common mosaic virus* (BCMV) host differentials which included race Durango lines UI-123, UI-34, UI-114, UI-31, and UI-35, to see if any of the well characterized potyvirus resistance genes provided cross protection to BGYMV. In the glasshouse tests, NW-395, UI-114, UI-31, and UI-35 exhibited variable resistant reactions ranging from no symptoms to a low to intermediate level for mosaic chlorosis, plant stunting, and flower abortion. Interestingly, all are resistant to BCTV except UI-123, which had the most susceptible BGYMV symptoms of the eight race Durango lines tested. The field reaction of these same lines was difficult to determine because they lacked adaptation to the tropical environment in Guatemala. Morales and Singh (1991) further investigated the breeding potential for UI-114, UI-31, and UI-36 in diallel crosses with Andean and race Mesoamerican lines to estimate GCA for reaction to BGYMV in the GH. Porrillo Sintetico had the most resistant scores for foliar yellowing, plant dwarfing, flower abortion, and pod formation and UI-114 was the next least susceptible of the eight parental lines. Given this promising performance for UI-114, it was crossed with ICA Pijao (which has the resistance source Porrillo Sintetico in its background) to study inheritance of resistance and breed for resistance in an interracial RIL population (Morales and Singh, 1993) using mechanical inoculation in the GH. Eleven of the RILs were symptomless for mosaic and disease incidence, suggesting that each of the parents with intermediate reactions contributed different and complementary genes, which together improved resistance. This supported the benefits of crossing among resistance sources from different gene pools. This approach resulted in resistant lines A429 (*bgm-1* source) and DOR 303 (*bgm-2* source). It may be beneficial to further investigate the contribution of Durango race materials like UI-114 for improving resistance to BGYMV, specifically when combined with race Mesoamerican sources of resistance like Porrillo Sintetico and ICA Pijao. Although general lack of adaptation of race Durango germplasm in the tropical climates in Central America could limit their utilization for improving BGYMV resistance, the exception is USRM-20 released in Nicaragua, which may provide an adapted race Durango source to mobilize additional resistance. Recently, *bgm-1* was mobilized in pinto beans for tropical production (Beaver et al., 2020b).

Andean sources of resistance can also be useful for improving BGYMV resistance in Middle American germplasm (Singh et al., 2000) and in the large-seeded red kidney, yellow, and red mottled market classes grown in the Caribbean and Panama. Badillo likely with *bgm-2* based on its pedigree (Beaver et al., 1999) and high level of resistance in the BASE_120, Royal Red (Singh et al., 2000), and PR1146-123 with similarly high level of resistance in the BASE_120 and with PvNAC1 marker indicating presence of *bgm-1*, provide useful sources of resistance in an Andean background.

To identify other resistance genes to combine with *bgm-1*, QTL linkage mapping in two RIL populations and GWAS in three populations were performed. The DX RIL population was re-genotyped with SNP markers, and the genomic intervals for BGY.4.1 and BGY7.1 QTL were narrowed. GWAS in the BASE_120 population detected peaks for these same two QTL. The validation of the QTL in multiple populations encouraged the mapping of additional SNPs and indels spanning the QTL intervals. Subsequent haplotyping revealed the S04_2531038 SNP marker in a putative candidate gene *Phvul.004G022000* with potential for MAS of BGY4.1. The S04_2531038 SNP is a missense variant located in the second exon of the CYP82C4 (cytochrome P450, family 82, subfamily C, polypeptide 2 and 4) gene. Members of the cytochrome P450 superfamily are involved in multiple metabolic pathways influencing plant growth and development, and abiotic and biotic stress responses (Xu et al., 2015). Similar cytochrome P450 family proteins in *A. thaliana* were upregulated upon geminivirus AC2/C2 protein infection (Babu et al., 2018). Simulated MAS showed that the presence of the BGY4.1 linked marker (S04_2531038) did not influence resistance in the CIAT breeding population. Another concern for BGY4.1 is the proximity to a genomic hotspot for anthracnose, halo blight, rust, and other disease resistance loci associated with LRR gene clusters (Meziadi et al., 2016). Thus, MAS for BGY4.1 could displace other important resistance genes that are in repulsion (*trans*) linkage.

The BGY7.1 QTL, first noted by Miklas et al. (1996) in the DX population, had poor resolution; thus, it was not pursued for MAS. The re-genotyped DX population increased the genomic resolution for BGY7.1 and revealed a greater effect of the QTL on resistance (40.6% PVE) than previously thought. But, GWAS in the BASE_120 detected a less significant peak for BGY7.1. Nonetheless, a similar interval for BGY7.1 overlapping the *Bct-1* gene conditioning resistance to BCTV and BDMV was observed in both DX and BASE_120 populations. A tightly linked SNP marker S07_2970381 within the *Phvul.007G036300* (exonuclease V) candidate gene (described above) for *Bct-1* works well for MAS of *Bct-1* in snap beans and Andean dry beans, but the same marker is nearly ubiquitous in germplasm of the Middle American gene pool which is predominately grown in the BGYMV affected regions of Central America (Soler-Garzón et al., 2018). Additional markers, SNP S07_2966197 and indel S1137_407 adjacent *Phvul.007G036300* candidate gene, were mapped in the DX population and proposed for MAS of BGY7.1. S07_2966197 was the most polymorphic in the DDP (58%), but their BGYMV phenotype was not assessed to examine association of the marker with resistance. S1137_407 exhibits some potential for MAS of BGY7.1 in race Mesoamerican lines as observed in the BASE panels, but additional validation of the QTL-linked marker is warranted. This region of Pv07 is of high importance for resistance to geminiviruses, given *Bct-1* conferring qualitative resistance to BCTV, and quantitative resistance to BDMV is within the BGY7.1 genomic interval. In addition, resistance to *Bean leaf crumple virus* (BLCV), a newly described geminivirus of common bean in Colombia (Carvajal-Yepes et al., 2017), maps within 2 Mb of the BGY7.1 QTL (unpublished).

The BGY8.1 QTL was observed in the DS population as a minor effect QTL that enhanced resistance when combined with *bgm-1* but did not provide resistance on its own. BGY8.1 exhibited the same epistatic interaction of enhanced resistance in combination with *bgm-1* but no effect by itself when analyzed by GWAS in the CIAT breeding population. Although validated in two different populations using two different methods, the interval for BGY8.1 was relatively broad (∼500,000 bp). Therefore, the SNP marker (S08_9202267) chosen for BGY8.1 in this study should be considered a reference point to further narrow the interval for the QTL and search for selectable markers. For example, the S08_9202267 marker did not contribute to enhanced resistance when combined with *bgm-1* in the BASE_120 population.

Interestingly, the BGY8.1 QTL was effective in the CIAT and DS populations that were screened for disease reaction in El Salvador but not in the BASE_120 population that was screened in Honduras. Similarly, the BGY4.1 QTL was effective in BASE_120, and DX populations screened in Honduras or Puerto Rico but not in the CIAT population screened in El Salvador. Could BGY4.1 and BGY8.1 be exhibiting a differential interaction with the prevalent strains in each country whereby they are effectively conditioning resistance against certain strains but not all? Distinct differences among BGYMV strains in the region have been observed (Garrido-Ramirez et al., 2000). Or perhaps different evaluations (1–9 score in El Salvador vs. incidence in Honduras) or different severity or timing of the epidemics contributed to a differential effect for the QTL. Nonetheless, further evaluation of the QTL-linked markers is necessary to ensure their utility for MAS. Meanwhile, MAS for *bgm-1*, using the new PvNAC1 candidate gene marker, followed by phenotypic selection in the field, is the recommended breeding strategy for obtaining common bean cultivars with high levels of resistance to BGYMV.

## Data Availability Statement

The datasets presented in this study can be found in online repositories. The names of the repository/repositories and accession number(s) can be found in the article/ Supplementary Material.

## Author Contributions

AS-G conducted the genomics analyses. AO conducted the GWAS. JB, SB, and JCR coordinated phenotyping. RL, PM, and QS generated SNP data. JDL and EM conducted genotyping and haplotyping. BR and SB provided a breeding population and conducted analyses. PNM led this team effort. AS-G and PNM wrote the manuscript. All authors contributed to the article and approved the submitted version.

## Conflict of Interest

The handling editor RP and the author PNM declare that they are affiliated with the INCREASE consortium on genetic resources in legumes. The remaining authors declare that the research was conducted in the absence of any commercial or financial relationships that could be construed as a potential conflict of interest.

## Footnotes

1 https://phytozome-next.jgi.doe.gov/info/PvulgarisUI111_v1_1

2 https://phytozome.jgi.doe.gov/pz/portal.html#!info?alias=Org_Pvulgaris

3 https://phytozome-next.jgi.doe.gov/info/PvulgarisUI111_v1_1

4 http://www.geneious.com

5 https://phytozome.jgi.doe.gov/pz/portal.html#!info?alias=Org_Pvulgaris
