# Supplementary material for: NAC Candidate Gene Marker for bgm-1 and Interaction With QTL for Resistance to Bean Golden Yellow Mosaic Virus in Common Bean
Source: Front Plant Sci. 2021 Mar 25;12:628443. doi: 10.3389/fpls.2021.628443 (PMC8027503; doi:10.3389/fpls.2021.628443)
Supplement: Supplementary file 1 [file Presentation_1.pdf]

Supplementary Figure 1 (3 slides).
DOR 364 / XAN 176 (DX) 79 RILs
395 SNPs markers, 2 SCARs and 1 indel

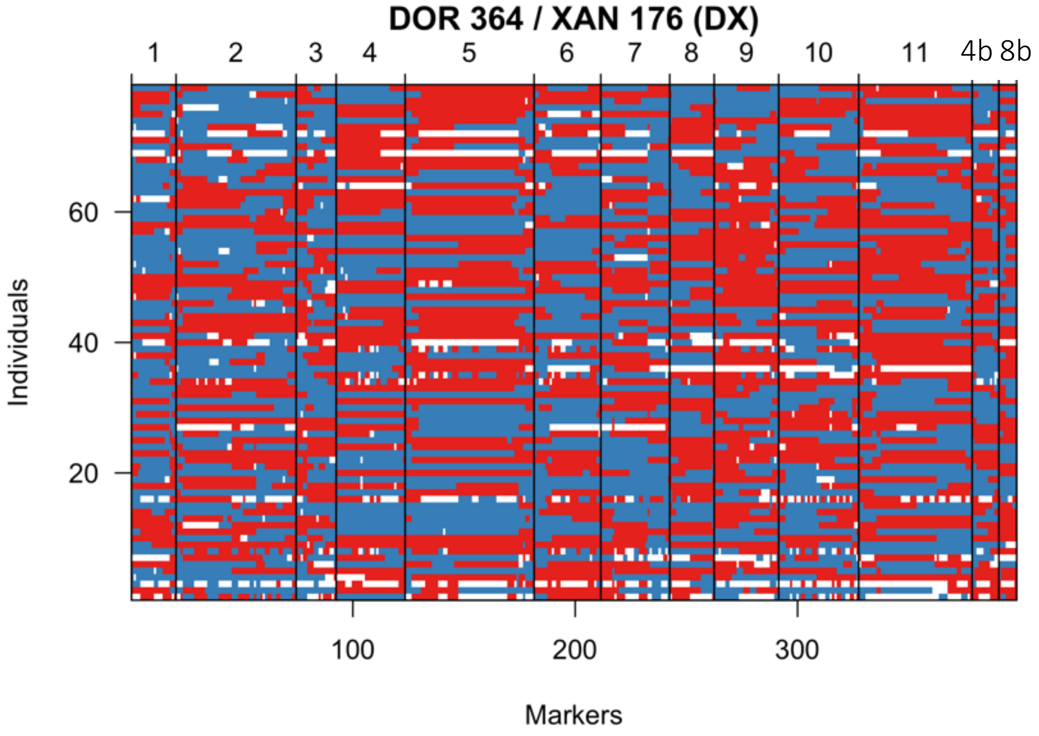

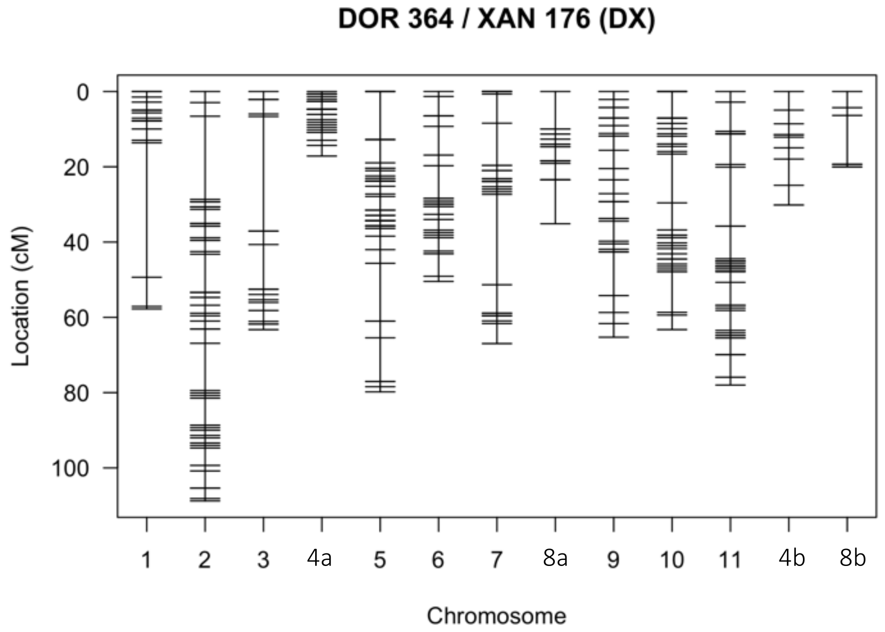

Chr	No. Markers	Length	ave.spacing	max.spacing
Pv01	20	57.8	3	35.7
Pv02	54	108.8	2.1	22.1
Pv03	18	63.3	3.7	30.4
Pv04a	31	17.1	0.6	2.8
Pv04b	12	30.2	2.7	7
Pv05	58	79.8	1.4	15.4
Pv06	30	50.5	1.7	8.7
Pv07	31	67	2.2	24
Pv08a	20	35.1	1.8	11.7
Pv08b	8	20	2.9	13
Pv09	29	65.3	2.3	11.6
Pv10	36	63.3	1.8	13
Pv11	51	78	1.6	15.7
overall	398	736	1.9	35.7

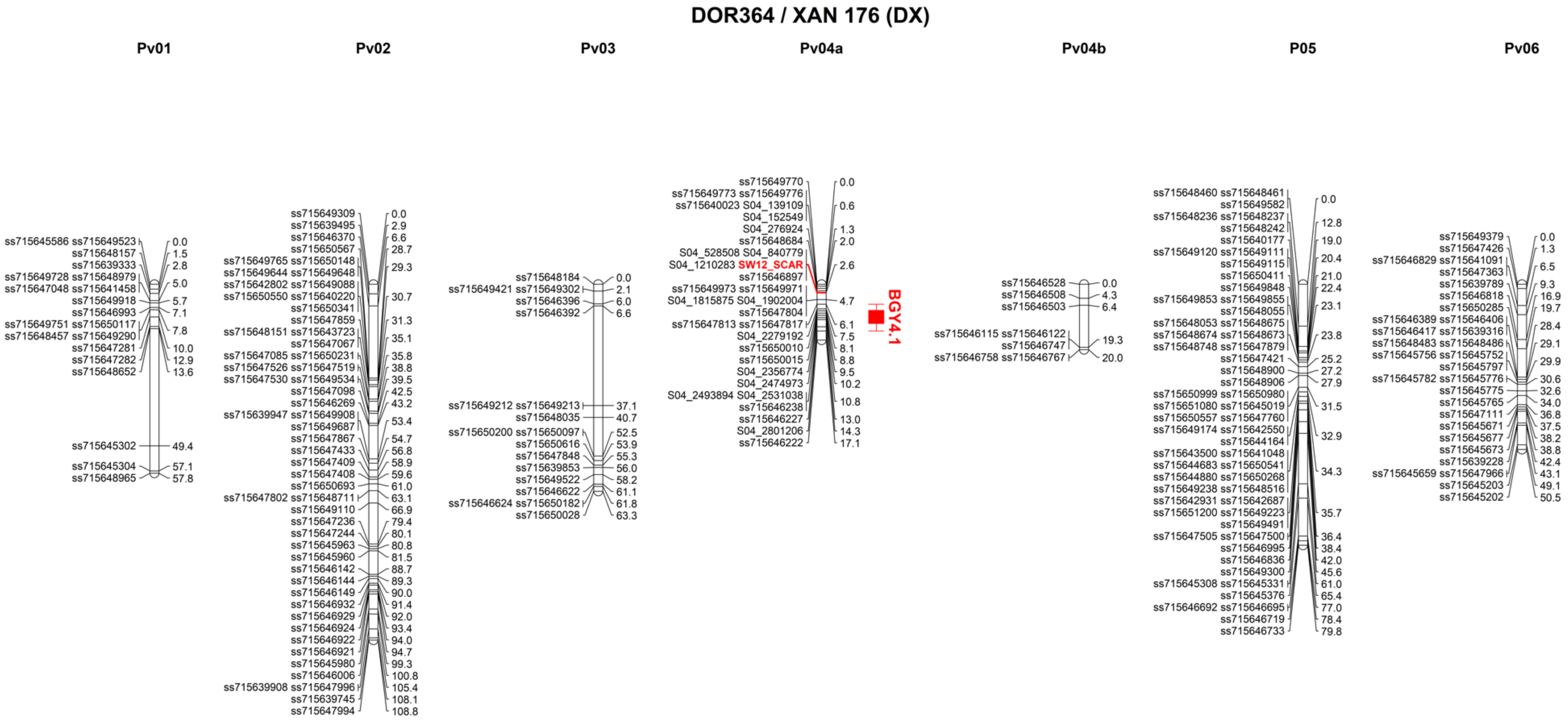
DOR364 / XAN 176 (DX)
Pv01
Pv02
Pv03
Pv04a
Pv04b
P05
Pv06
SW12_SCAR
BGY4.1

DOR364 / XAN 176 (DX)

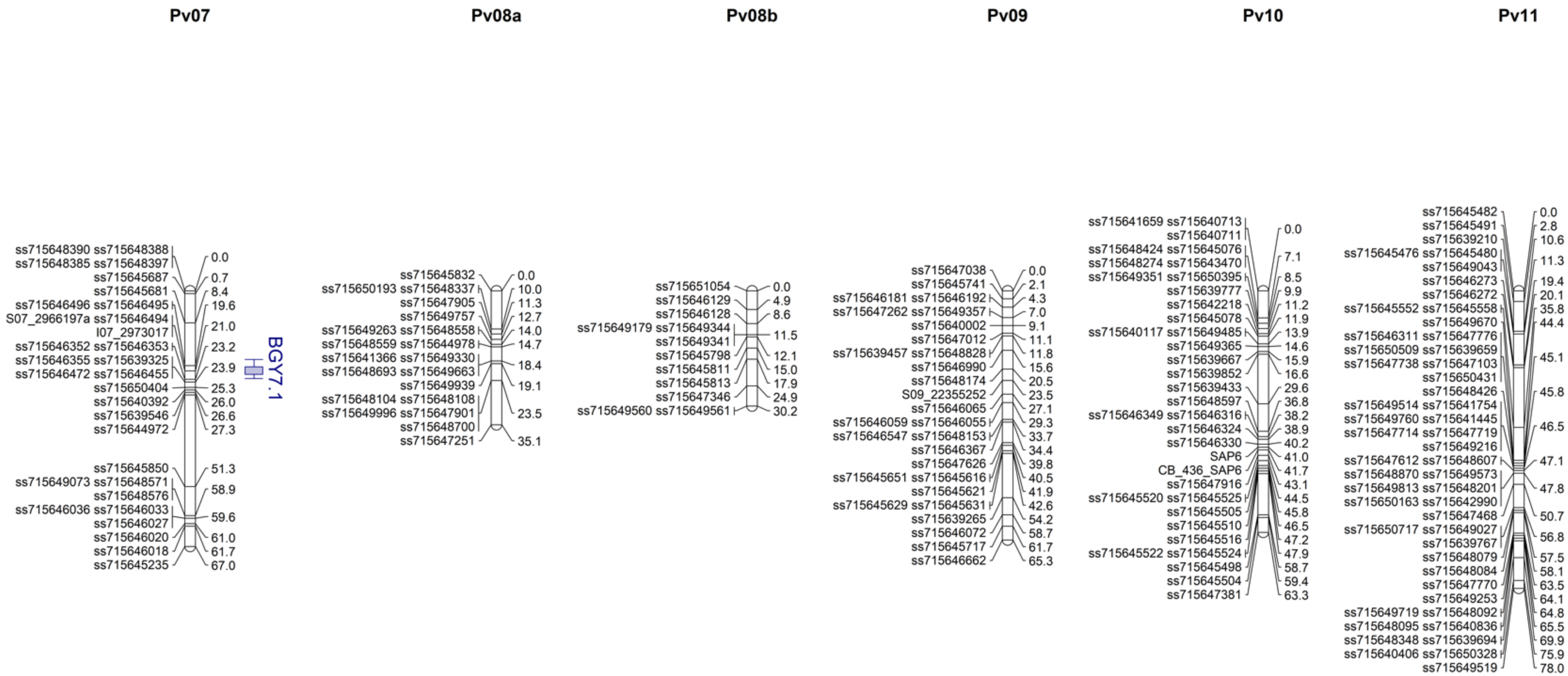

Supplementary Figure 2 (3 slides).
DOR476 / SEL1309 (DS) 96 RILs
585 SNPs markers

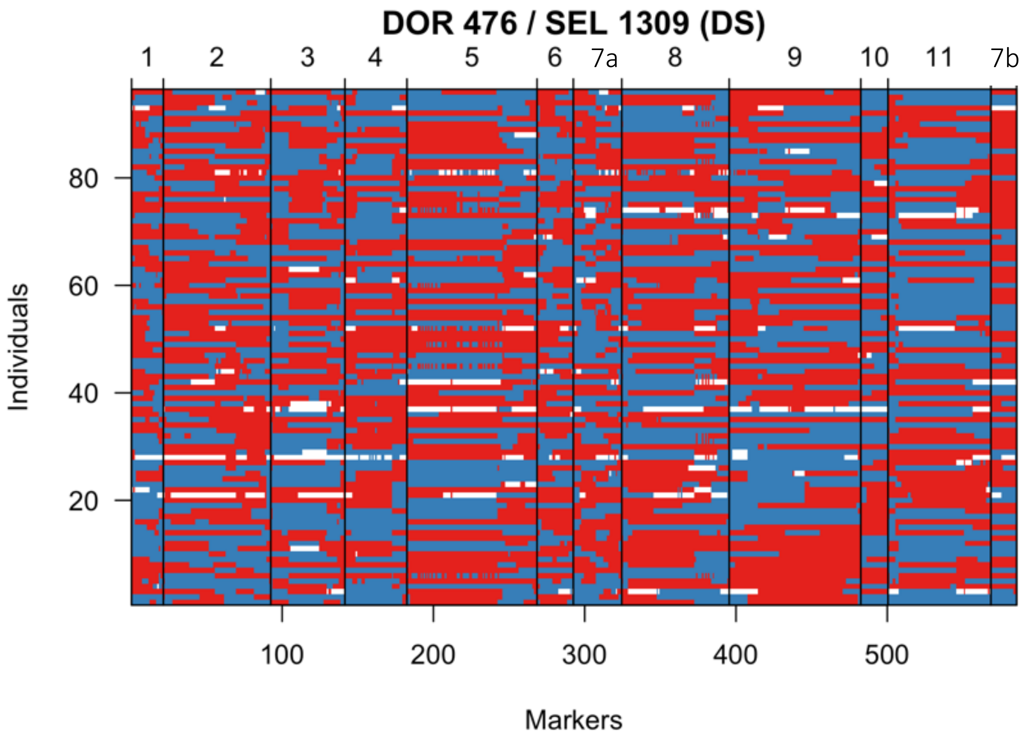

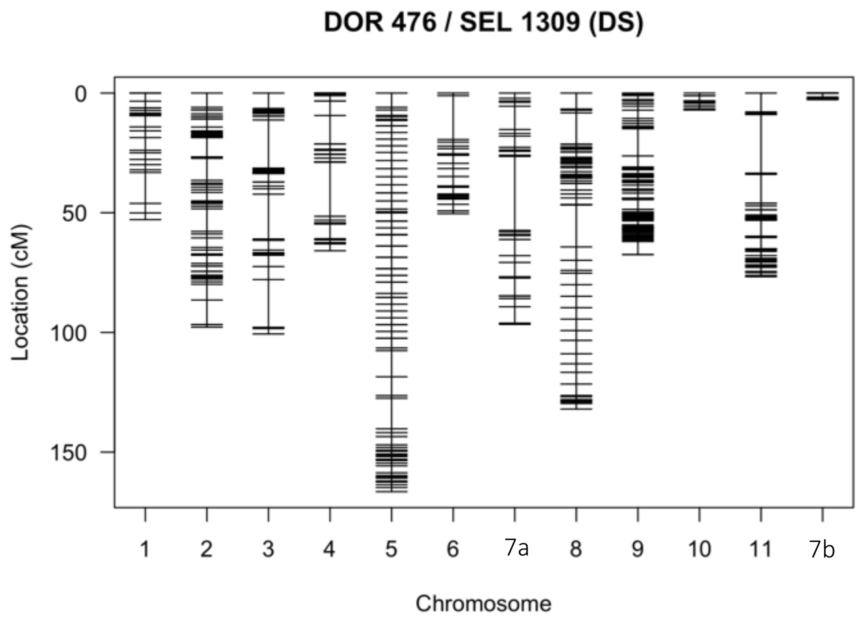

Chr	No. Markers	Length	ave.spacing	max.spacing
Pv01	21	52.9	2.6	12.9
Pv02	71	97.8	1.4	10.3
Pv03	49	100.6	2.1	20.1
Pv04	41	65.9	1.6	22.6
Pv05	86	166.5	2	12.8
Pv06	24	50.4	2.2	18.4
Pv07a	32	96.6	3.1	30.9
Pv07b	17	2.7	0.2	1.7
Pv08	71	132	1.9	17.6
Pv09	87	67.5	0.8	11.4
Pv10	18	7	0.4	2.2
Pv11	68	76.7	1.1	24.5
overall	585	916.7	1.6	30.9

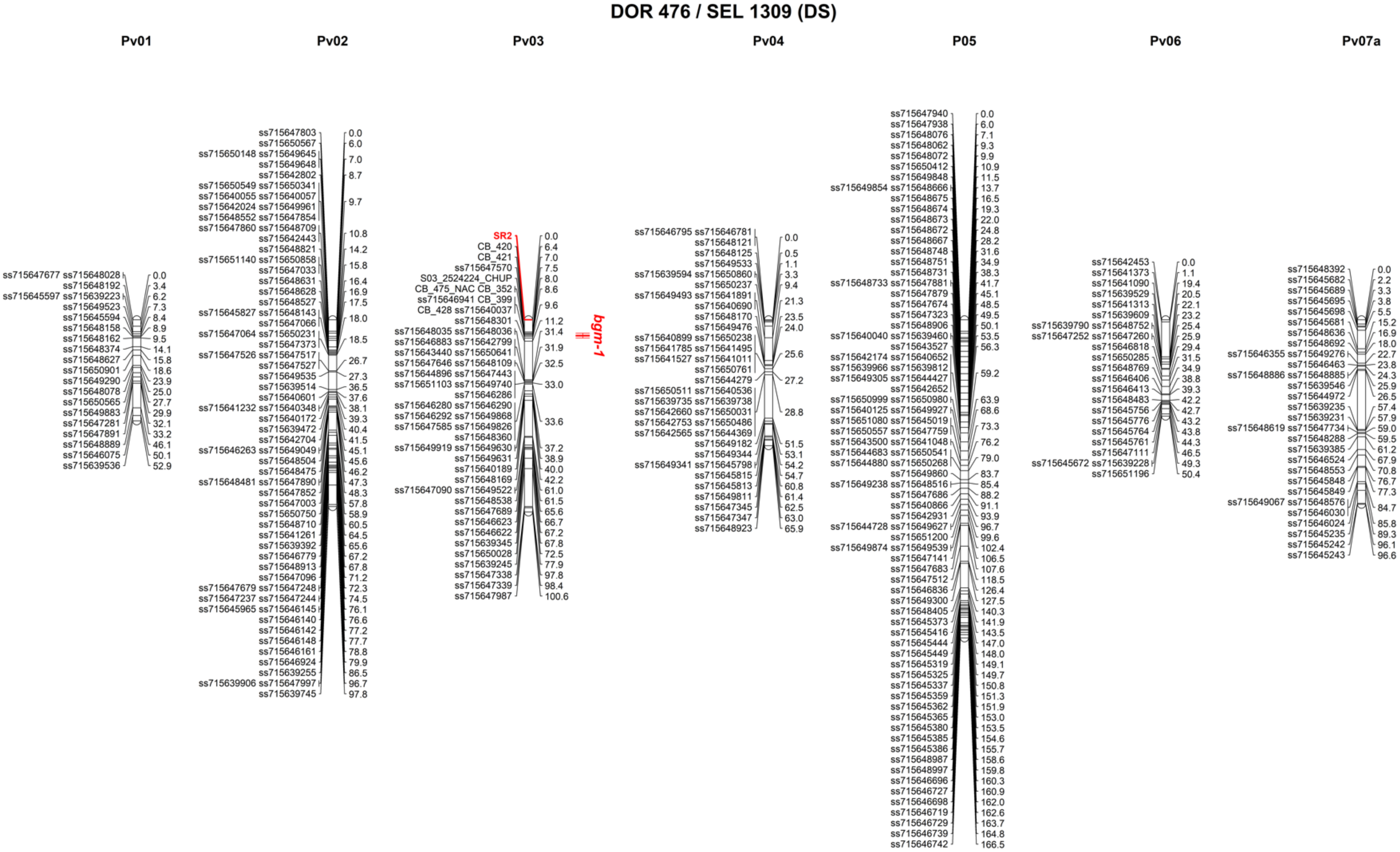
DOR 476 / SEL 1309 (DS)
Pv01
Pv02
Pv03
Pv04
P05
Pv06
Pv07a
SR2
bgm-1

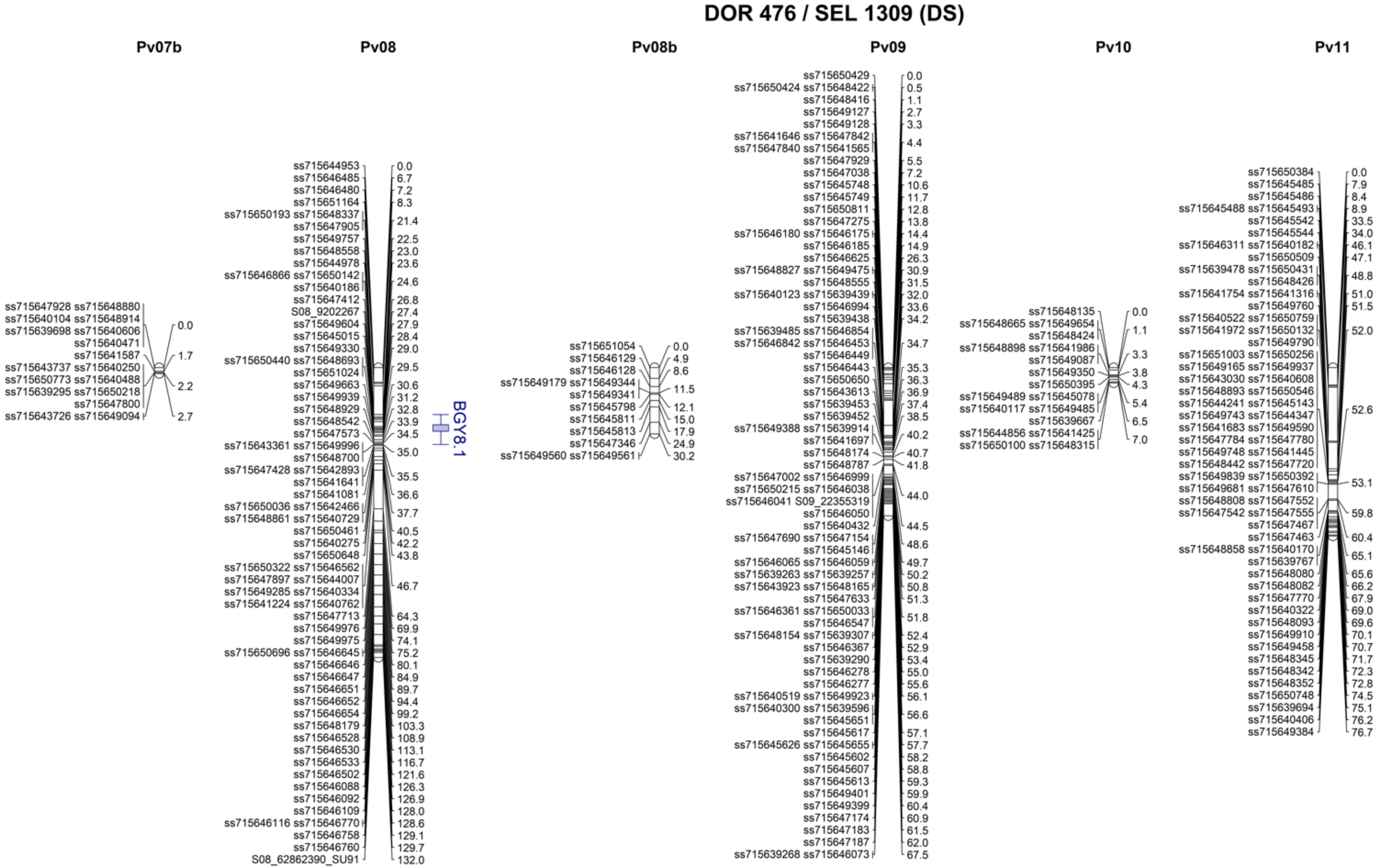
DOR 476 / SEL 1309 (DS)
Pv07b
Pv08
Pv08b
Pv09
Pv10
Pv11
BGY8.1
