# Supplementary figures and images for: NAC Candidate Gene Marker for bgm-1 and Interaction With QTL for Resistance to Bean Golden Yellow Mosaic Virus in Common Bean
Source: Front Plant Sci. 2021 Mar 25;12:628443. doi: 10.3389/fpls.2021.628443 (PMC8027503; doi:10.3389/fpls.2021.628443)

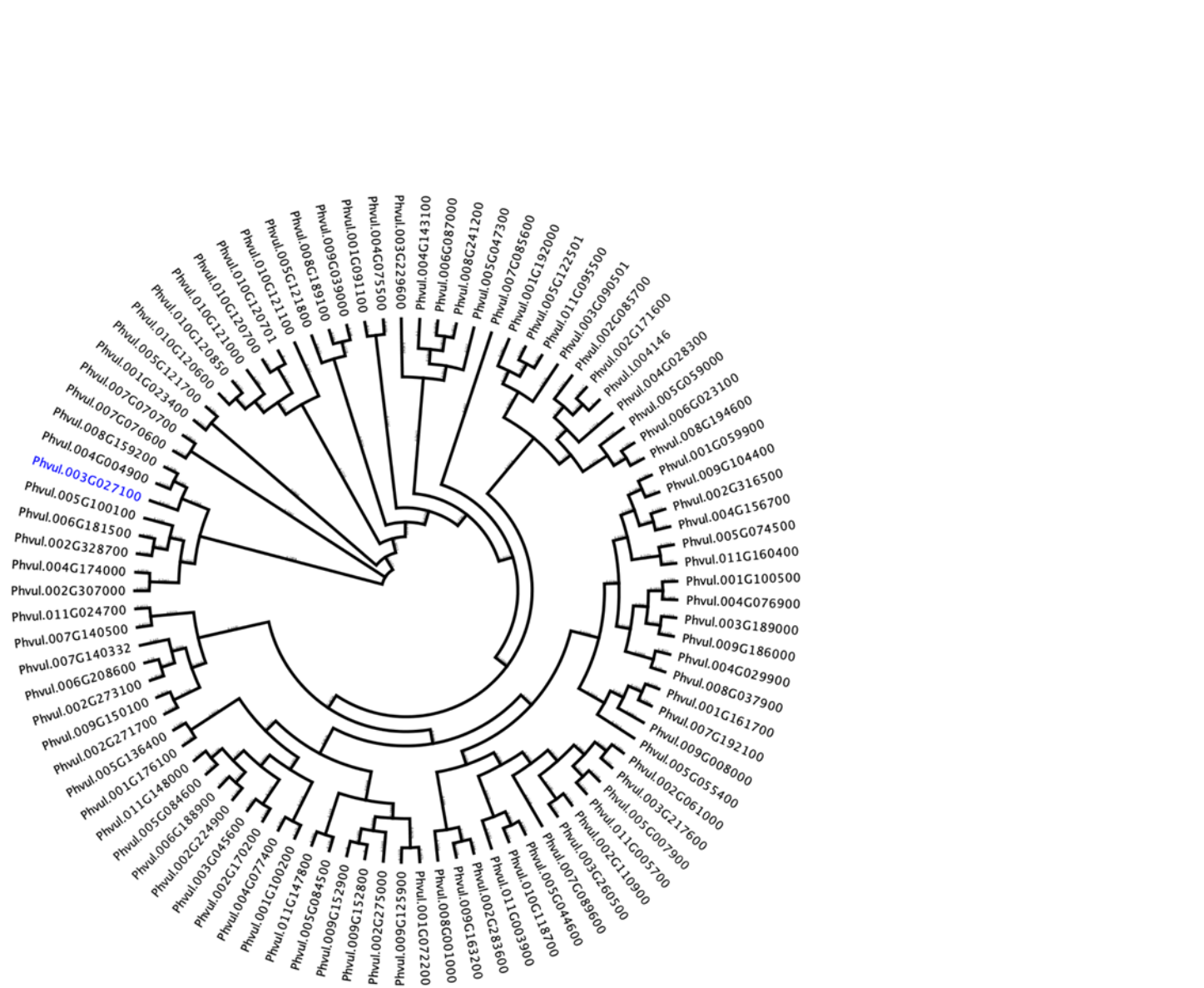

**Fig. S3** Neighbor joining tree for NAC genes in *P. vulgaris*.

Supplement: Supplementary file 2 [file Presentation_3.pdf]
